# Workforce Crisis in Radiology in the UK and the Strategies to Deal With It: Is Artificial Intelligence the Saviour?

**DOI:** 10.7759/cureus.43866

**Published:** 2023-08-21

**Authors:** Sadhana Kalidindi, Sanjay Gandhi

**Affiliations:** 1 Radiology, University of Bristol, Bristol, GBR; 2 Radiology, North Bristol NHS Trust, Bristol, GBR

**Keywords:** uk radiology, nhs, ai, artificial intelligence, radiology workforce

## Abstract

Radiology has seen rapid growth over the last few decades. Technological advances in equipment and computing have resulted in an explosion of new modalities and applications. However, this rapid expansion of capability and capacity has not been matched by a parallel growth in the number of radiologists. This has resulted in global shortages in the workforce, with the UK being one of the most affected countries. The UK National Health Service has been employing several conventional strategies to deal with the workforce situation with mixed success. The emergence of artificial intelligence (AI) tools that have the potential to increase efficiency and efficacy at various stages in radiology has made it possible for radiology departments to use new strategies and workflows that can offset workforce shortages to some extent. This review article discusses the current and projected radiology workforce situation in the UK and the various strategies to deal with it, including applications of AI in radiology. We highlight the benefits of AI tools in improving efficiency and patient safety. AI has a role along the patient’s entire journey from the clinician requesting the appropriate radiological investigation, safe image acquisition, alerting the radiologists and clinicians about critical and life-threatening situations, cancer screening follow up, to generating meaningful radiology reports more efficiently. It has great potential in easing the workforce crisis and needs rapid adoption by radiology departments.

## Introduction and background

In modern healthcare, radiology plays a central and pivotal role in the management of a majority of patients. The current grave workforce shortages in radiology in the UK are having a serious negative impact on the healthcare sector and are threatening optimal patient care. The current massive shortfall of 33% in the radiology workforce is expected to increase to 44% by the year 2024 [1]. Nearly 71% of clinical directors of UK radiology departments feel that they do not have a sufficient number of radiologists to deliver safe and effective patient care [1]. Further, the gross financial impact secondary to the workforce shortages in the form of outsourcing, insourcing, and locum costs will have a further deleterious effect on health services [2]. This already grave situation has been further compounded by the COVID-19 pandemic.

The advent of advanced deep learning tools coupled with the availability of high-quality datasets has made radiology a particularly suitable field of medicine for the development of a wide range of artificial intelligence (AI) algorithms that can impact the full spectrum of the functioning of radiology services [3]. The last few years have seen a substantial number of use cases being established and the emergence of several tools that are available for real-world clinical adoption [4]. AI is also likely to be able to play a very important role in screening, personalized medicine, and population health [5]. Over the last few years, there have been extensive and concerted efforts to bring clarity around governance for AI in medical imaging, including information governance as well as matters related to regulation.

## Review

Radiology is one of the youngest specialties in medicine and one that has been undergoing constant evolution due to its intricate links to technology, which keeps advancing at a rapid pace.

Between 1895 when Roentgen discovered X-rays and the 1960s, the majority of examinations were radiographic, fluoroscopic, angiographic, and ultrasound-related. The initial decades in the evolution of radiology involved high doses of radiation and severely invasive procedures. The *golden era* of radiology started in the 1970s when the invention of the CT scanner opened the possibility of cross-sectional imaging [6]. The last 50 years have seen ground-breaking advances in both CT and MRI technology, which have led to a rapid increase in the applications of imaging.

The increasing demand for imaging over the last few decades has caused exponential growth in the specialty. This is seen in multiple forms [7,8], including an increase in the number of scanners, the number of studies performed on each scanner, the number of images per patient, and the complexity of the studies. The number of studies requested per patient has also increased along with the percentage of referrals that are not adequately evidence-based. There has also been an increase in the range of non-reporting work performed by radiologists, e.g., multidisciplinary team meetings (MDTMs).

**Figure 1:**
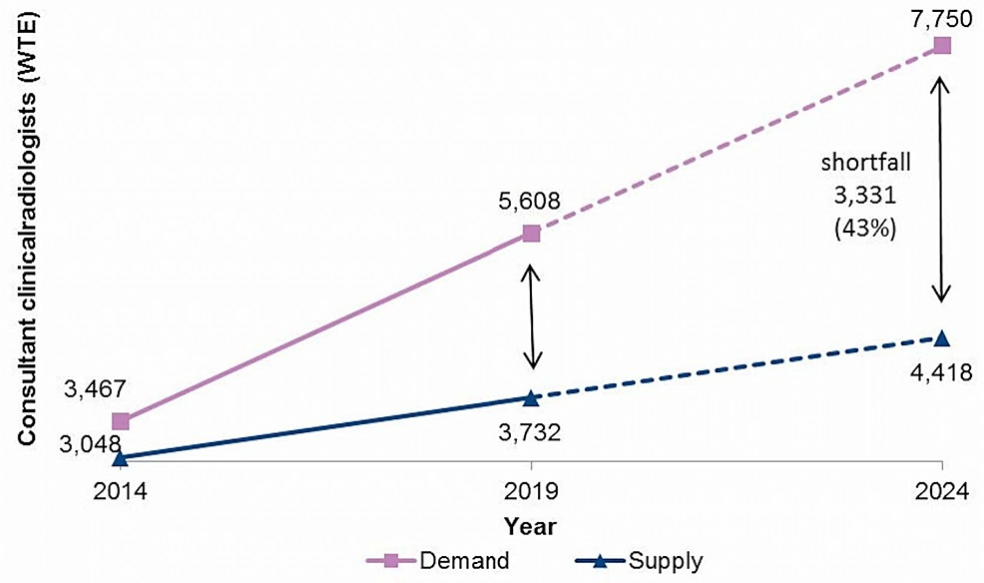
Estimated supply and demand of whole-time equivalent consultant radiologists in the UK. Source: Royal College of Radiologists. Clinical Radiology UK Workforce Census 2019 report [1] (Reproduced with permission).

To meet the current demand and provide safe service, the National Health Service (NHS) needs 5,608 whole-time equivalent radiologists. According to the Royal College of Radiologists (RCR) 2019 workforce census, there was a shortage of 1,876 radiologists. This gap is set to widen further in the coming years due to increasing demand and increasing numbers of consultant radiologists leaving each year (Figure 1). In 2019, just 1% of UK Trusts were able to meet their reporting requirements within consultant radiologists’ contracted hours [1].

**Figure 2:**
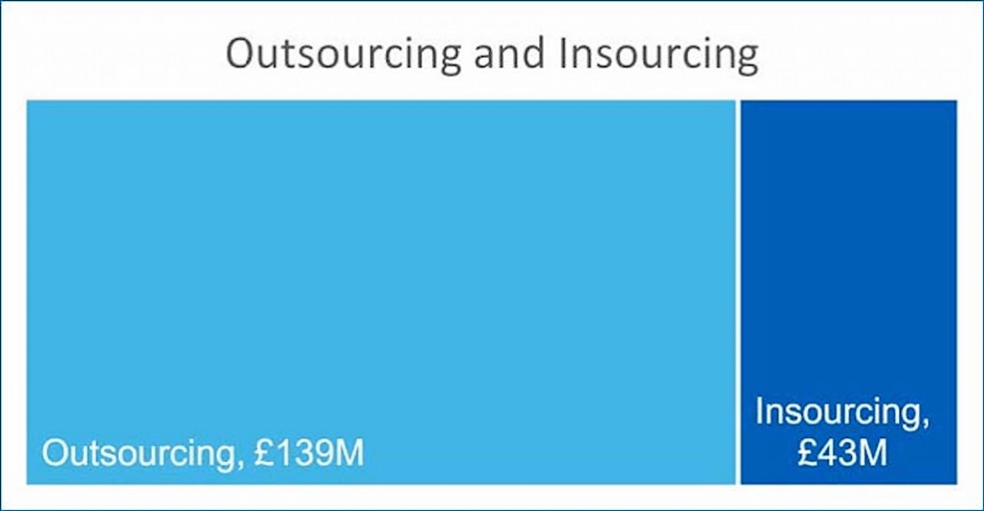
Outsourcing and insourcing. Source: National Imaging Data Collection 2017/18, NHS Improvement [2].

The annual expenditure by the NHS to deliver imaging services is £2 billion. More than two-thirds of this spend is toward staffing and more than £160 million is spent on non-substantive pay [2]. This includes expenditure for locums and insourcing and outsourcing to teleradiology companies and other providers (Figure 2).

**Figure 3:**
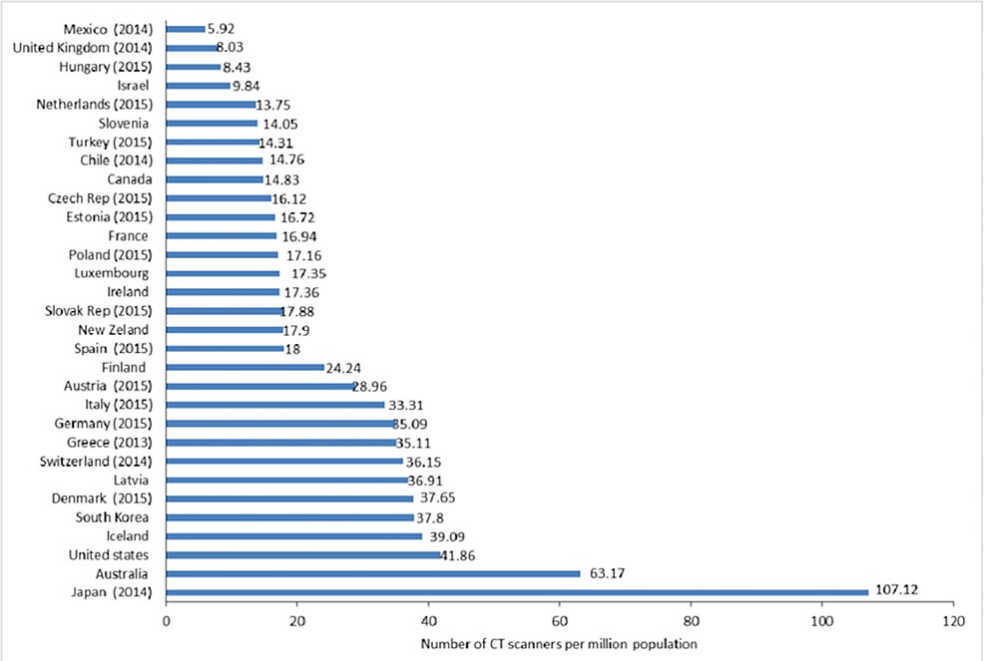
Number of CT scanners in the UK. Source: Lara et al. [10] (This article is available under the Creative Commons CC-BY-NC license and permits non-commercial use, distribution, and reproduction in any medium).

The UK has one of the lowest numbers of CT scanners, ranking second lowest among 31 countries in the number of scanners per million population (Figure 3). The situation with the number of MRI scanners is slightly better, but, overall, the UK lags significantly. A recent report by Sir Mike Richards [9] recommends that the CT scanning capacity should be increased by 100% over the next five years. This will result in a significant increase in the volume of studies and workload.

Impact of COVID-19

**Figure 4:**
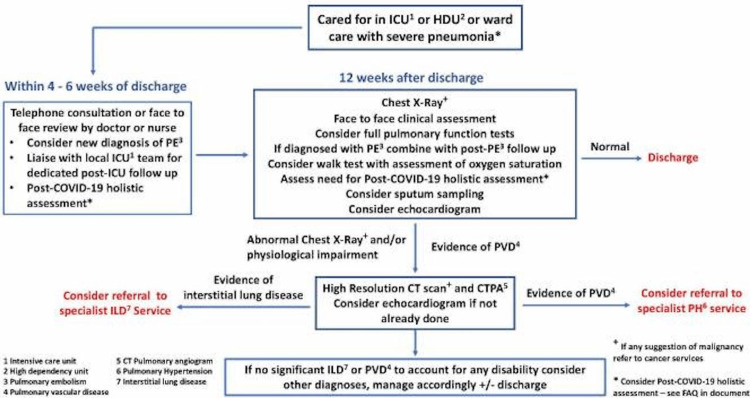
Suggested algorithm for following up patients with severe COVID-19 pneumonia. Source: George et al. [12] (Reproduced with permission).

Like all aspects of healthcare, the COVID-19 pandemic has caused significant disruption and modification of imaging provision across the country. There was a significant reduction in elective requests both from primary care and secondary care initially, as well as a reduction in the throughput due to the protective measures. This created a large backlog of imaging requests. Restoration of imaging services is a complex issue linked to multiple factors, including baseline demand and the catch-up of the requests kept on hold due to the pandemic [11]. In addition, the long-term sequelae, including pulmonary sequelae, for patients who recovered from severe COVID-19 are still unclear. Follow-up algorithms suggested including a CT of the thorax in some of these patients [12]. Figure 4 is an example of one such algorithm. At the time of writing, more than 300,000 patients were discharged following hospitalization for COVID-19. Following up on these patients for interstitial lung disease or vascular disease might add a serious additional burden on the imaging services. Furthermore, such a large volume of lung scans might also lead to the detection of incidental nodules, which might require follow-up.

In summary, there have been chronic and ever-increasing shortages in the radiology workforce in the UK, which is likely to be seriously compounded further by increasing demand and the direct and indirect impact of the COVID-19 pandemic. Therefore, the next five years are likely to be one of the most challenging periods ever for imaging services in the UK. Urgent action both through conventional routes as well as the adaption of innovative and disruptive methods and technology is required to be able to provide safe patient care.

Conventional strategies employed to deal with the situation

Increase in Training Capacity

For a specialty that has seen chronic shortages, an increase in training numbers has been the obvious first solution. However, radiology training departments have been too stretched to meet the demand for increased capacity. In 2005, the RCR and the Department of Health set up three radiology academies to increase the training numbers and provide effective specialist training. These academies have resulted in a two-to-five-fold increase in training numbers in the respective schemes. Since 2013, Health Education England has invested £2.43 million per year in the three original radiology academies [13].

In 2016, RCR published a 10-year vision [14] consisting of several initiatives, including setting up several new academies and expanding e-learning and simulation-based learning.

Skill Mix in Radiology

The concept of the skill mix in radiology to address the shortages was conceived in the later 1990 [15]. This led to the development of innovative roles and effective career pathways for radiographers and encouraged team working within radiology departments. Radiographers have successfully taken on advanced practitioner roles that include reporting.

However, Sir Mike Richards’ report [9] estimates that in the next five years, in addition to 2,000 radiologists, there will be a need for 500 reporting radiographers/advanced practitioners in England alone. This restricts the degree to which further expansion of the skill mix can be employed.

Retention Strategies

About 20% of the current consultant radiologist workforce is expected to retire in the next five years [1]. This is not only a loss of highly experienced and skilled staff but also a loss of significant teaching capacity. Retaining some of these consultants either on a full-time or part-time basis will substantially help in easing the situation.

Working From Home

The COVID-19 pandemic has brought reporting from home into the mainstream even for routine reporting. Many hospitals have invested in the rapid deployment of equipment for home reporting [11]. An investment was also made in solutions that allow remote participation in the MDTMs. This expansion in adaption and acceptance of home reporting could be used to potentially improve retention and increase flexibility and productivity.

International Recruitment

Recruitment of radiologists from overseas has been increasing over the last few years. In 2019, 40% of clinical radiologists recruited underwent their specialist training overseas [1]. It is almost certain that COVID had a major impact on overseas recruitment in 2020 and is likely to continue to do so in 2021. Increasing global shortages also add to the uncertainty.

Newer and innovative strategies

Networking

Imaging departments organizing themselves and delivering services through networks is shown to be one of the ways of dealing with the problems of workforce shortages and aging equipment. The benefits include better utilization of radiologist and radiographer capacity, sharing backlog reporting, increasing levels of radiographer reporting, etc. This has multiple benefits to patient care and is shown to reduce reporting times and missed diagnoses [2]. Four early adapter imaging networks were commissioned by NHS Improvement in 2018. Based on the experiences gained from these networks and other clinical networks, NHS England and Improvement published a national approach in “Transforming imaging services in England: a national strategy for imaging networks” [2].

Community Diagnostic Hubs

Traditionally, imaging services in the UK have been delivered through departments in the acute hospital setting. This has limited the throughput and efficiency of the departments and resulted in suboptimal patient experience. Separation of acute imaging from elective imaging will have a significant positive impact on the services and will result in more effective delivery [10]. The recent report by Sir Mike Richards advised the creation of Community Diagnostic Hubs away from acute hospitals with operating procedures that will enable rapid and seamless scanning services to be offered to patients. The patients served in the hubs could include both direct general practitioner referrals as well as outpatient hospital referrals.

Artificial Intelligence

AI has the potential to drive a disruptive innovation that can reduce the burden on imaging departments and improve the speed, efficiency, accuracy, and safety of services. Although still evolving, there are a good number of applications that are ready for adoption primarily for triaging, prioritization, and screening [4,5]. Given the current grave situation with the workforce, radiology departments across the country must take a proactive approach toward the adoption of AI applications and expand further as more of them become available for safe clinical use. The remainder of this article discusses the basics of the technology, the current state, the future, and the potential for a positive impact on patient care.

Artificial intelligence, machine learning, and deep learning: the definitions

Artificial Intelligence

The term AI was first coined by John McCarthy in 1956 [16]. The simplest definition of AI is “the capability of a machine to imitate intelligent human behavior” (Merriam-Webster). Since then, AI has gone through periods of boom and bust with “AI Winters” caused by various factors, including limitations in computing power, funding, etc.

Machine Learning

Machine learning is a form of AI that concerns the development of computer programmes that can find patterns within complex datasets and produce intellectual predictions without explicit human programming [17]. Machine learning can be supervised or unsupervised. In supervised learning, the model is given labeled input data that is associated with a specific output. In unsupervised learning, the model learns to find patterns in data with an unknown outcome [4].

Deep Learning

**Figure 5:**
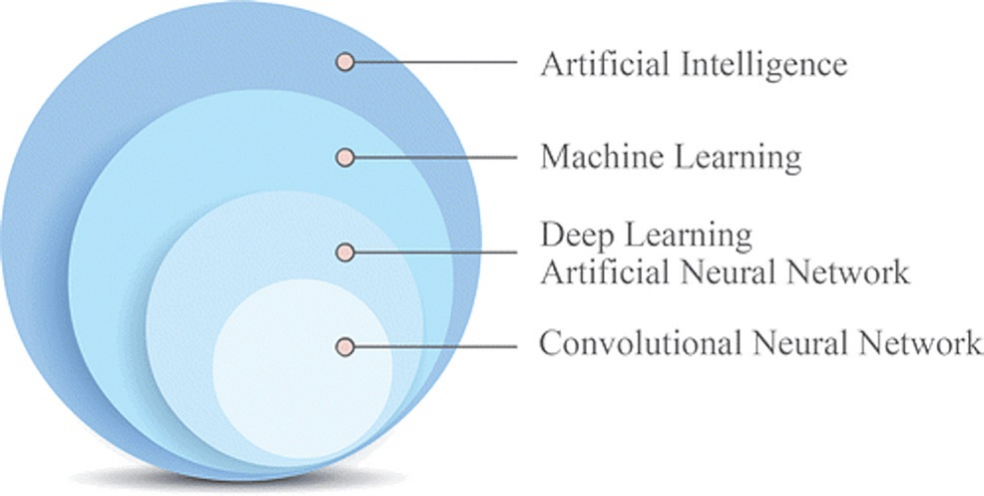
Venn diagram representation of convolutional neural networks in the artificial intelligence hierarchic terminology. Source: Soffer et al. [19] (This article is available under the Creative Commons CC-BY-NC license and permits non-commercial use, distribution, and reproduction in any medium).

This is a class of machine learning that consists of models which are based on multilayered artificial neural networks which are somewhat inspired by biological neural systems. Although neural networks have been used for a long time, in recent years, there have been rapid advances in their development because of the availability of large, labeled datasets, a substantial reduction in the cost of parallel computing hardware, and improvement in the training architectures [18]. Early neural networks had less than five layers. Deep learning uses networks that have many layers, usually more than 20. Convolutional neural networks (CNNs) are artificial neural networks that explicitly assume that the inputs are images (Figure 5) [19]. In the last few years, CNN technology has become a leading innovation in computer vision. Deep learning models are used in voice recognition, facial recognition, automatic language translation, and driverless cars.

Deep learning and radiology

The availability of large datasets made radiology a particularly suitable specialty for the application of deep learning in healthcare [4]. AI has the potential to assist in every stage in the process of provision of radiology service and thereby increase the productivity and efficiency of radiologists, reduce errors, and increase patient safety.

Imaging Request

Clinical Decision Support (CDS) tools have been developed based on the RCR’s evidence-based best clinical practice guidelines called iRefer. CDS tools can help general practitioners and hospital clinicians in picking up the correct imaging modality for the most common clinical scenarios. CDS can also reduce unnecessary clinical tests.

Image Acquisition

Ongoing research has shown that AI has the potential to reduce radiation dose in CT by creating high-resolution images from noisy low-dose scans. It could also predict the appearance of a contrast-enhanced CT from a non-contrast study [20].

Normal Versus Abnormal

AI algorithms that can be primarily used to segregate normal studies from abnormal studies have been developed [21,22]. The main areas of interest were chest X-rays and CT of the brain. The chest X-ray model can be used to prioritize reporting abnormal X-rays and reduce the delayed diagnosis of serious abnormalities caused by reporting backlogs.

Worklist Prioritization and Triaging

Radiologist worklists could be reprioritized using AI algorithms that can detect possible acute abnormalities, flag such studies, and move them to the top of the reporting lists. This can reduce turnaround times for the detection of acute findings, such as intracranial hemorrhage and pneumothorax, showing a tangible benefit to patient care [23].

Assistance in Reporting

A large proportion of the radiologists’ time, in modern practice, is spent on tedious and repetitive tasks which do not require rational interpretation or judgment. AI models can take over these tasks and free up radiologists’ time to perform the clinical interpretation of the findings, other complex clinical tasks, clinicoradiological discussions, procedures, etc. The speed of reporting and productivity from radiologist sessions will also increase, which will be of significant help in the current workforce situation. Examples of the tasks where AI can assist include the detection of lung nodules, segmentation, and lesion measurement, as well as comparison with previous scans in case of follow-up scans for incidental findings or post-treatment scans for cancer [4.18].

Following Up on Actionable Findings

Natural language processing (NLP) is the process of extracting and structuring natural language into formal computer representations [24]. NLP can help in the management of cases where follow-up is recommended in reports by automatically generating alerts for subsequent examinations or procedures [25].

Screening

Imaging-based screening studies such as breast cancer screening and potential targeted lung cancer screening cause a very high degree of burden on radiologists. AI can be very effective in reducing this burden and at the same time reducing missed cancers. AI models can exceed human performance in breast cancer detection. In an example of the potential in a clinical setting [26], an AI system when evaluated on a curated large dataset from the UK and US has shown an absolute reduction of 5.7% and 1.2% (USA and UK) in false positives and 9.4% and 2.7% in false negatives, respectively. When the double reading system as used in the UK was simulated, the model showed non-inferior performance and reduced the workload of the second radiologist by 88%.

Population Health

As we move into the era of preventive and personalized medicine, pre-emptive detection and quantification of important incidental findings that are not normally reported but have population health significance become very important. Examples include deep learning models that can assess bone mineral density from unenhanced abdominal CT scans [27] and those that can quantify coronary calcium scores from routine unenhanced non-gated chest CT scans [28].

The landscape in the UK

The Royal College of Radiologists

In its position statement on AI dated July 20, 2018 [29], the RCR stated that it “believes that AI potentially represents one of the most fundamental changes in medical care since the inception of the NHS, and strongly welcomes the introduction of appropriately regulated and governed uses of AI-related technologies to enhance clinical practice.” It concludes that far from making the clinical radiologist and clinical oncologist of the future redundant, the use of AI will help standardize many aspects of clinical care, optimize processes, and allow greater use of clinical data to inform best practices and outcomes.

NHSX

The NHSX is responsible for the delivery of the tech plan and brings together teams from DHSC and NHS E&I to drive digital transformation and lead policy, implementation, and change [6]. This document states that an investment of £250 million is going into the creation of NHS AI Lab “to focus on supporting innovation in an open environment where innovators, academics, clinicians and others can develop, learn, collaborate and build technologies at scale to deliver maximum impact in health and care safely and effectively.” It will be run collaboratively by NHS and the Accelerated Access Collaborative. It will closely evaluate mechanisms to overcome the pain points in the regulation of AI. Currently, the regulatory landscape shows suboptimal coordination between the bodies involved. Attempts are also underway to address data regulation to ensure that data innovation is not hindered while also ensuring patient confidentiality requirements are fully met.

## Conclusions

The current workforce shortages in radiology are seriously threatening the delivery of safe patient care in radiology, and this situation is forecast to worsen over the coming years. Coupled with increasing demand and the projected increase in the equipment base, this will endanger the sustainability of radiology services. Several initiatives to ease the situation have been undertaken, but these are unlikely to resolve the crisis on their own. The impact of the COVID-19 pandemic has resulted in a significant additional burden.

Recent advances in deep learning technology have made it possible for AI-based algorithms to be developed that can positively impact all aspects of radiology services and make them more efficient, effective, and productive. Several use cases for AI-assisted diagnostic tools are rapidly being established and will soon be available for radiologists to adapt. An increased speed in the adaption of AI in radiology departments across the UK can be of help in ensuring that services remain safe and sustainable.

## References

[1] The Royal College of Radiologists (2020). Clinical Radiology UK Workforce Census 2019 Report. https://www.rcr.ac.uk/system/files/publication/field_publication_files/clinical-radiology-uk-workforce-census-2019-report.pdf.

[2] Denton E, McCaughey H (2019). Transforming Imaging Services in England: A National Strategy for Imaging Networks. https://webarchive.nationalarchives.gov.uk/ukgwa/20210401201200/https:/improvement.nhs.uk/documents/6119/Transforming_imaging_services.pdf.

[3] Lee LI, Kanthasamy S, Ayyalaraju RS, Ganatra R (2019). The current state of artificial intelligence in medical imaging and nuclear medicine. BJR Open.

[4] (2019). What the radiologist should know about artificial intelligence - an ESR white paper. Insights Imaging.

[5] Joshi I, Morley J (2019). Artificial Intelligence: How to Get it Right. Putting Policy Into Practice for Safe Data-Driven Innovation in Health and Care. https://transform.england.nhs.uk/media/documents/NHSX_AI_report.pdf.

[6] (2023). History of radiology - British Institute of Radiology. https://www.bir.org.uk/useful-information/history-of-radiology.aspx.

[7] Clinical Imaging Board (2015). CT Equipment, Operations, Capacity and Planning in the NHS. Radiologists.

[8] Kane B, Luz S, O'Briain DS, McDermott R (2007). Multidisciplinary team meetings and their impact on workflow in radiology and pathology departments. BMC Med.

[9] Richards M (2020). Diagnostics: Recovery and Renewal. Report of the Independent Review of Diagnostic Services for NHS England. NHS England.

[10] Lara A, Osorio M, Olvera B, Villafañez YO, García R, Rivera T (2019). Importance of patient radiation protection in computed tomography procedures. J Physics.

[11] Dickson J (2023). Impact of COVID-19 on imaging services in the UK. https://hospitalhealthcare.com/latest_issue_article/impact-of-covid-19-on-imaging-services-in-the-uk/.

[12] George PM, Barratt SL, Condliffe R (2020). Respiratory follow-up of patients with COVID-19 pneumonia. Thorax.

[13] (2023). Health Education England. National Review of Radiology Academies. https://www.hee.nhs.uk/sites/default/files/documents/Review%20of%20radiology%20academies%20FINAL.pdf.

[14] Royal College of Radiologists (2016). Radiology Training 2016-2026: A Vision and a Solution. Radiologists.

[15] Denton E, Wivell G (2008). Skill mix and teamwork in imaging departments: redesigning the clinical team. HealthManagement.

[16] Marr B (2023). The key definitions of artificial intelligence (AI) that explain its importance: Bernard Marr. https://www.forbes.com/sites/bernardmarr/2018/02/14/the-key-definitions-of-artificial-intelligence-ai-that-explain-its-importance/?sh=41b036424f5d.

[17] Chartrand G, Cheng PM, Vorontsov E (2017). Deep learning: a primer for radiologists. Radiographics.

[18] Yamashita R, Nishio M, Do RK, Togashi K (2018). Convolutional neural networks: an overview and application in radiology. Insights Imaging.

[19] Soffer S, Ben-Cohen A, Shimon O, Amitai MM, Greenspan H, Klang E (2019). Convolutional neural networks for radiologic images: a radiologist's guide. Radiology.

[20] Harvey H, Topol EJ (2020). More than meets the AI: refining image acquisition and resolution. Lancet.

[21] (2023). Artificial intelligence shows potential for triaging chest X-rays. https://www.rsna.org/news/2019/january/ai-for-chest-x-rays.

[22] Jha S (2020). Value of triage by artificial intelligence. Acad Radiol.

[23] O'Neill TJ, Xi Y, Stehel E, Browning T, Ng YS, Baker C, Peshock RM (2021). Active reprioritization of the reading worklist using artificial intelligence has a beneficial effect on the turnaround time for interpretation of head CT with intracranial hemorrhage. Radiol Artif Intell.

[24] (2023). Natural language processing in radiology | Laboratory of Quantitative Imaging and Artificial Intelligence (QIAI). https://rubinlab.stanford.edu/node/323.

[25] Pons E, Braun LM, Hunink MG, Kors JA (2016). Natural language processing in radiology: a systematic review. Radiology.

[26] McKinney SM, Sieniek M, Godbole V (2020). International evaluation of an AI system for breast cancer screening. Nature.

[27] Yasaka K, Akai H, Kunimatsu A, Kiryu S, Abe O (2020). Prediction of bone mineral density from computed tomography: application of deep learning with a convolutional neural network. Eur Radiol.

[28] van Assen M, Martin SS, Varga-Szemes A (2021). Automatic coronary calcium scoring in chest CT using a deep neural network in direct comparison with non-contrast cardiac CT: a validation study. Eur J Radiol.

[29] (2023). RCR position statement on artificial intelligence. https://www.rcr.ac.uk/posts/rcr-position-statement-artificial-intelligence.

